# Campho-Phenique

**Published:** 1889-09

**Authors:** J. Foster Flagg

**Affiliations:** Philadelphia, Pa.


ARTICLE VII.

### CAMPHO-PHENIQUE.

BY J. FOSTER FLAGG, D. D. S., PHILADELPHIA, PA.

The rapidly-developing importance of this peculiar combination of carbolic acid and camphor impels me to a presentation of its especial claims as, probably, the most remarkable medicament which has ever been offered in connection with dental therapeutics.

When it is known that it is a notable germicide, an efficient antiseptic, a non-irritant, a decided local anæsthetic, non-poisonous, insoluble in water or glycerine, does not discolor or stain, is possessed of an agreeable odor and not disagreeable taste, and maintains an unchanged integrity, it will at once be recognized as wonderfully adapted to a large proportion of all dento-pathological conditions, from sensitivity of dentine, through the varying conditions of pulp-irritation, pulp-devitalization, pericemental irritation, alveolar abscess, and caries or necrosis of contiguous osseous structure, and that thus it must rank as one of the most, if not *the most valuable* polychrest which dentistry possesses.

During the past session of the college with which I am connected (since September, 1888) I have availed myself of the extended opportunities afforded for a decisive clinical record of this material, and the results have been so gratifying that it is with much satisfaction that I present its claims to recognition and urge a prompt acceptance of the many benefits it has to bestow.

Where cotton is indicated as a wedge, and especially where marked sensitivity of dentine exists in connection with such cavities between teeth, both the discomfort attending separating and the pain attendant upon subsequent preparation of cavities are largely, and frequently completely, abrogated.

In cases of pulp-irritation, even of severe grade, its application, upon cotton, will almost invariably demonstrate its high rank as a " pain obtundent."

In devitalization of pulps its use as the menstruum for the arsenic and acetate of morphia in our " devitalizing paste" seems to have already given evidence of its value as a local anæsthetic in that connection. As a disinfectant of tissue surrounding pulp cavities and canals which have contained putrescent pulps it has made an excellent record, and has proven itself, by its variety of peculiarly acceptable attributes, to be one of the very best applications we have ever had for the purpose.

As a medicament, or ingredient of medicaments, for canal-dressings, either temporary or *permanent*, upon cotton, its combined characteristics of *antisepsis* and *insolubility* must command favorable recognition.

As an antiphlogistic in the earlier stages of sthenic pericementitis, applied upon the gum with small pads of muslin and renewed with *only desirable infrequency*, it has oftentimes been able to produce the attempted resolution; and, in cases where this was found impossible, to largely mitigate the suffering attending the induction of suppuration.

As an antipyogenic, used by injection into fistulæ, either in full strength or diluted by fluid or viscid cosmoline or lanolin, it has produced eminently satisfactory results in some markedly discouraging cases.

It will thus be seen that, from the dental stand-point, campho-phenique is a medicine which it behooves us to test thoroughly; that if it shall be found to perform even a portion of the good offices which it so largely promises, suffering humanity shall promptly rejoice over this additional assuager of some of its many ills.

Although intimation of other uses than those pertaining strictly to dentistry might here be regarded as irrevelant yet so many phases of trouble, such as wounds (cut or contused), burns, sprains, intolerable itchings, etc., are so decidedly relieved by applications of campho-phenique (either pure or diluted), that I feel sure that those unfortunates who may chance, through such mention, to find relief from these inflictions cannot but feel grateful for this information.

Campho-phenique is stated by its manufacturers, The Phenique Chemical Company of St. Louis, to be a definite chemical compound, having a formula $C_8 H_{11}O$, and thus, "for obvious reasons," it has had given to it the name under which it is presented to the healing professions.—*The Dental Cosmos.*

---

ARTICLE VIII.

